# Supplementary material for: A Template-Based Approach for Guiding and Refining the Development of Cinnamon-Based Phenylpropanoids as Drugs
Source: Molecules. 2020 Oct 11;25(20):4629. doi: 10.3390/molecules25204629 (PMC7587175; doi:10.3390/molecules25204629)
Supplement: Supplementary file 1 [file molecules-25-04629-s001.pdf]

Supplementary Material

Table S1: Significant downregulated KEGGs pathways identified by DAVID following exposure to five cinnamon-based phenylpropanoids ($p < 0.05$).

	**Term: Genes**	**p-value (Benjamini)**
Chlorogenic acid	Cytokine-cytokine receptor interaction: FASLG, TNFSF14, CXCL11, IL11, FLT3LG, CCL3L1, CCL3L3, CXCR6, XCR1, RTEL1, CSF2RA, TNFRSF17, TNFRSF14, CCNL2, VEGFB, AMH, TNFRSF10B, INHBE, IFNB1, CCR3, VEGFA, CCR2, IL12A, CCL1, CCL3, CXCL5, TNFRSF25, CCR1, CSF1, CX3CL1, CCL7, CCL24, TNFRSF1B, IL12RB1, CCL21, FIGF, EPO, IL4, IL18R1, FLT1, TGFBR1, EDA2R, HGF, TNFSF8, KDR, LEP, GH2, CCL13, EPOR, XCL1, IFNA16, XCL2	2.43×10^{5}
	Neuroactive ligand-receptor interaction: OPRM1, THRA, GRIK1, DRD2, GRIK2, TACR2, TACR1, GABRB1, LPAR4, GRIK5, FPR1, PRSS1, GNRHR, FPR2, EDNRA, AGTR2, LTB4R, PRSS2, CNR1, S1PR4, CALCRL, TAAR5, GABRE, PTGER1, GABRG3, C5AR1, PTGER3, PTGER4, GABRA6, GABRA5, GRM1, PLG, LEP, CRHR1, GH2, GRM3, SSTR2, CHRM3, GRIA1, MC2R, P2RX2, TBXA2R, GHSR, HTR2C, TSHR, LHB, GLP1R, OPRD1	9.68×10^{5}
	Hematopoietic cell lineage: IL4, CR1, CD8B, CSF1, FCER2, GYPA, ITGA2, IL11, GP9, FLT3LG, CD38, CD19, DNTT, GP1BB, CD22, EPOR, CSF2RA, CD14, THPO, EPO, HLA-DRA, ITGA2B	9.29×10^{4}
Cinnamaldehyde	Cytokine-cytokine receptor interaction: IL6ST, IL21R, IL19, TNFSF15, CXCR3, IL15, CXCL11, TGFB1, IL11, FLT3LG, CXCL10, CCR10, XCR1, RTEL1, CSF2RA, IL21, CCNL2, VEGFB, CCR8, AMH, TNFRSF10C, IFNB1, PDGFRA, EDA, CXCL5, TNFRSF25, CSF1, IFNW1, CNTFR, CX3CL1, CCL5, TNFRSF4, CCL4, CCL27, CCL24, CCL25, CCL23, IFNA6, IFNA5, FIGF, EPO, AMHR2, IL2RA, FLT4, TGFBR2, EDA2R, HGF, TNFSF8, CCL17, KDR, LEP, GH1, CCL13, CNTF, TNFSF11, PRLR, EPOR	1.46×10^{7}
	Neuroactive ligand-receptor interaction: THRA, TRPV1, DRD2, LPAR1, ADORA1, GCGR, VIPR2, EDNRB, HTR1A, HRH2, LTB4R, CNR1, P2RY1, MC3R, TAAR5, GABRP, GABRE, PTGER4, GZMA, GABRA6, GRIA3, GRM1, PLG, LEP, P2RX5, GH1, GRM3, SSTR2, P2RY11, PRLR, CHRM3, GRM8, GRIA1, HTR7, MC2R, AVPR1A, TBXA2R, ADRA1A, HTR2C, LHB, GLP1R, CTSG, MTNR1A	1.22×10^{2}
	Focal adhesion: TLN1, MYL2, TNC, ITGB4, ELK1, MYL10, SRC, VCL, PAK6, BCL2, COMP, SOS2, COL11A2, FIGF, COL11A1, AKT3, AKT2, COL4A4, PRKCA, FLT4, ACTN2, MAPK10, HGF, COL4A6, KDR, VEGFB, LAMA2, LAMA4, PPP1CA, ITGA7, PDGFRA, RELN, MAPK8, COL1A1, PARVB	1.98×10^{2}
	Cell adhesion molecules (CAMs): HLA-DQB1, PVR, ICAM1, MAG, PTPRC, CD8A, CD8B, CLDN3, CLDN6, CLDN5, NFASC, CLDN11, CLDN14, PDCD1LG2, SDC3, CD86, PVRL1, HLA-DPA1, VCAN, HLA-DOA, HLA-DOB, SELPLG, CD226, ICOSLG, SPN	3.88×10^{2}
Cinnamic acid	Cytokine-cytokine receptor interaction: LEPR, IL21R, TNFSF15, IL15, CXCL11, TGFB1, FLT3LG, CXCL10, ACVR1B, CXCR5, CCL3L1, CCL3L3, CXCR6, LTB, RTEL1, CD40, IL21, CCNL2, AMH, PPBP, CCR2, VEGFA, EDA, CSF3, CCL3, CXCL5, TNFRSF25, CCR1, CCL5, CCL24, IL12RB1, IFNA6, IFNA5, CD27, EPO, IL18R1, FLT1, IL2RA, EDA2R, HGF, CCL15, TNFSF8, GH2, CCL13, TNFSF11, CNTF, EPOR, BMPR1B, IFNA17	1.60×10^{3}
	Hematopoietic cell lineage: CSF3, CR1, IL2RA, CD3D, CD8B, FCER2, ITGA2, ITGA3, CD1E, GP9, FLT3LG, GP5, CD37, CD44, FCGR1A, CD2, CD22, CD4, EPOR, THPO, EPO, HLA-DRA	3.03×10^{3}
	Neuroactive ligand-receptor interaction: CGA, DRD1, THRA, ADORA3, TACR3, CYSLTR1, GRIK2, TACR2, LEPR, LHCGR, LPAR4, PRSS1, LPAR1, FPR2, VIPR2, GHRHR, HTR1A, LTB4R, PRSS2, S1PR4, CNR1, NPFFR1, GABRD, GABRE, PTGER3, PTGER4, GRIN1, GRM1, PLG, GH2, GRM5, CRHR2, SSTR2, SSTR1, GRIA1, HTR7, PTGDR, F2, MC2R, AVPR1A, HTR2B, HTR2C	2.67×10^{2}
	Calcium signaling pathway: ADCY1, GNA15, DRD1, ADCY2, TACR3, CYSLTR1, TACR2, LHCGR, ATP2B3, PTK2B, PDE1A, CHRNA7, PRKACA, CHRFAM7A, SLC8A1, PTGER3, GRIN1, CHP2, GRM1, GRM5, GNAL, PLCE1, SLC25A31, ATP2A3, PLN, HTR7, CACNA1G, AVPR1A, HTR2B, CACNA1C, HTR2C, MYLK	4.16×10^{2}
Ferulic acid	Cytokine-cytokine receptor interaction: ACVRL1, IL21R, TNFSF14, CXCR3, TNFSF12, CXCL11, CXCL10, FLT3LG, TGFB2, CCL3L1, CCR10, CCL3L3, CXCR6, IL15RA, XCR1, PRL, LTB, RTEL1, CSF2RA, TNFRSF17, TNFRSF14, CD40, CCR8, CCR7, IL12A, PDGFRA, PDGFRB, NGFR, TNFSF12-TNFSF13, CSF2, CCL3, CXCL5, CSF1, CXCL9, IFNW1, KIT, PF4V1, CCL4, CCL7, CCL24, IL17A, CCL23, IL10RA, IFNA8, FIGF, CD27, IL4, IL18R1, TNFSF4, FLT1, MET, HGF, CCL15, TNFSF8, GH2, LEP, GH1, CCL13, TNFSF11, BMPR1B, MPL	4.15×10^{9}
	Neuroactive ligand-receptor interaction: CSH1, OPRM1, THRA, GRIK2, LHCGR, LPAR3, VIPR2, EDNRA, S1PR1, LTB4R, S1PR4, GRIN2D, GLP2R, PRL, TAAR5, GRM1, CRHR1, GRM5, CHRM3, GRM8, SSTR1, MC2R, FSHB, TSHR, GLP1R, DRD2, PRSS1, FPR3, GCGR, HRH2, PRSS2, CNR1, PRSS3, ADRA2B, GABRA2, GLRB, PTH2R, GABRA3, GABRA6, GRIN1, GRIA3, NPY5R, GH2, LEP, GH1, P2RY10, GRIA1, P2RY14, P2RX2, AVPR1A, GHSR, LHB, HTR2C	6.33×10^{7}
	Hematopoietic cell lineage: IL4, CSF2, CR1, CD8B, CSF1, CD1C, KIT, ITGA4, CD1E, FLT3LG, CD38, CD44, DNTT, CD34, GP1BB, FCGR1A, MS4A1, CD22, CSF2RA, CD14, THPO, ITGA2B	8.69×10^{4}

	Intestinal immune network for IgA production: IL4, TNFRSF17, TNFSF12, CD40, ITGA4, HLA-DQA1, TGFB2, CD80, CCR10, IL15RA, HLA-DPA1, AICDA, TNFSF12-TNFSF13, HLA-DOB	4.30×10^{2}
Caffeic acid	Cytokine-cytokine receptor interaction: PDGFA, IL6ST, IL21R, TNFSF15, FASLG, TNFSF12, IL10, TGFB2, CCL3L1, IL1RAP, CCL3L3, CCR10, TPO, PDGFC, XCR1, PRL, LTA, CD40, IL11RA, PPBP, IL12B, TNFSF12-TNFSF13, EDA, CSF3, IL1R2, CCL3, IL1R1, CXCL5, CTF1, CSF1, CCL4, CCL7, CCL24, CCL25, IFNA2, IL17A, IFNA1, IL23A, IFNA6, IFNA5, FIGF, EPO, IL18R1, BMP2, IL2RA, MET, CCL19, HGF, GH1, CCL13, CNTF, CXCL14, XCL1, IFNA16	4.51×10^{6}
	Neuroactive ligand-receptor interaction: OPRM1, CGA, THRA, GABRB3, LHCGR, PTH1R, LPAR4, GRIK5, FPR1, VIPR2, HCRTR2, AGTR2, HTR1A, GRIN2C, PRSS2, CNR1, MC5R, GLP2R, PRL, TAAR5, HTR5A, GABRP, PTGER1, GLRB, PTGER3, GRIN1, GRIA3, GRM1, PLG, GRM5, GH1, GRM3, ADRB2, CHRM2, GRIA1, P2RX3, P2RY14, GIPR, P2RX2, AVPR1A, TBXA2R, GHSR, FSHB, LHB, CTSG, OPRD1	3.14×10^{4}
	Hematopoietic cell lineage: CSF3, IL1R2, IL1R1, CR1, IL2RA, CD3D, CD8B, CSF1, FCER2, GYPA, CD1B, ITGA2, CD1A, IL11RA, CD36, DNTT, CD44, ITGA5, TPO, THPO, EPO, HLA-DRA	6.53×10^{4}
	Autoimmune thyroid diseaseCGA, CTLA4, FASLG, CD40, HLA-DQA1, IL10, IFNA2, IFNA1, CD86, IFNA6, IFNA5, TPO, HLA-DPA1, IFNA16, HLA-DOA, HLA-DRA	8.35×10^{4}
	Cell adhesion molecules (CAMs): GLG1, CLDN18, CD8B, CLDN5, ITGB2, CDH5, PVRL1, CNTNAP2, HLA-DOA, SELPLG, SPN, PTPRC, PTPRM, ICAM2, CTLA4, NLGN3, NRXN1, CD40, PDCD1LG2, HLA-DQA1, NCAM1, CD86, CDH15, CLDN1, HLA-DPA1, ICOSLG, HLA-DRA	2.20×10^{3}
	Viral myocarditis: CAV1, MYH4, ITGB2, MYH7, CD40, HLA-DQA1, EIF4G1, CCND1, CD86, DMD, MYH11, SGCD, HLA-DPA1, HLA-DOA, MYH10, HLA-DRA	3.01×10^{2}

Table S2: Significant upregulated KEGGs pathways identified by DAVID following exposure to five cinnamon-based phenylpropanoids ($p < 0.05$).

	**Term: Genes**	**p-value (Benjamini)**
Chlorogenic acid	Neuroactive ligand-receptor interaction: CSH1, C3AR1, F2RL3, CCKAR, THRA, GABRB3, GRIK1, GABRB2, GRIK3, GLRA3, NPY2R, TRHR, GLRA2, GRIK5, GNRHR, GABBR2, SCTR, HCRTR2, EDNRB, ADRB3, HRH1, PTGIR, GALR1, GRIN2B, PRSS2, P2RY1, GALR2, GRID2, ADRA2A, ADRA2B, HTR1F, GABRQ, GABRA2, CCKBR, GRIN1, HTR4, GRIA4, PTGFR, GH1, CRHR2, GPR35, ADRB2, GRM7, PTGDR, TBXA2R, ADRA1A, TSHR, GLP1R	3.17×10^{4}
	Linoleic acid metabolism: CYP3A7, CYP2C19, CYP2C18, CYP2C9, JMJD7-PLA2G4B, PLA2G4A, AKR1B10, PLA2G2A, PLA2G1B, PLA2G2E, PLA2G4B, PLA2G5, PLA2G2F	1.45×10^{3}
	Arachidonic acid metabolism: CYP2C19, CYP2C18, CYP2C9, GPX1, CYP4A11, JMJD7-PLA2G4B, PLA2G4A, CYP4A22, PTGIS, ALOX15B, PLA2G2A, PLA2G1B, ALOX5, PLA2G2E, PLA2G4B, PLA2G5, ALOX12, PLA2G2F	1.42×10^{3}
	Hematopoietic cell lineage: IL4, IL3, IL1R1, IL9R, CR2, FLT3, CSF1, ANPEP, ITGB3, ITGA4, FLT3LG, GP5, CD36, FCGR1C, GP1BB, CD33, FCGR1A, MS4A1, TPO, CD5, IL1A, CD7	2.91×10^{3}
	Long-term depression: PPP2R1B, GNAO1, IGF1, ITPR1, JMJD7-PLA2G4B, PLA2G4A, PPP2CB, PLA2G2A, GRID2, PLA2G1B, RYR1, GUCY1B3, PLCB1, PLA2G2E, PLA2G4B, CACNA1A, PLA2G5, PLA2G2F	1.26×10^{2}
	Natural killer cell mediated cytotoxicity: KIR2DL5A, KLRK1, CD48, IFNA2, IFNA1, RAC2, IFNA5, FCGR3A, FCGR3B, PIK3R1, PIK3CG, ICAM2, HLA-C, NCR2, VAV1, NCR1, NCR3, FYN, ULBP1, LCK, IFNA13, KIR2DL3, KIR2DL2, KIR3DL1, KIR3DL2	2.38×10^{2}
	Vascular smooth muscle contraction: KCNMA1, ADCY2, MYLK3, CALD1, PRKCE, ITPR1, ACTG2, PRKCQ, CYP4A11, PTGIR, JMJD7-PLA2G4B, PLA2G4A, CYP4A22, PLA2G2A, PLA2G1B, ADRA1A, GUCY1B3, PLCB1, PLA2G2E, CACNA1D, PLA2G4B, PLA2G5, PLA2G2F	2.55×10^{2}
Cinnamaldehyde	Neuroactive ligand-receptor interaction: F2RL2, CSH1, OPRM1, CCKAR, F2RL3, THRA, GRIK1, LEPR, GLRA2, GNRHR, GABBR2, AGTR1, EDNRB, PTGIR, GRIN2B, NMUR1, GALR2, GRID2, PTGER3, CCKBR, HTR4, FSHR, CRHR2, GRM4, SSTR3, GRM8, GRM7, PTGDR, PTAFR, AVPR2, DRD1, CYSLTR1, DRD2, OPRK1, APLNR, CNR1, MAS1, GABRQ, GABRA2, GABRA1, PTH2R, GRIA3, GRIA4, PTGFR, GH2, GH1, P2RX7, TBXA2R, ADRA1B, ADRA1A, HTR2C, ADRA1D	1.45×10^{6}
	Calcium signaling pathway: CCKAR, DRD1, ADCY2, CYSLTR1, ADCY8, ATP2B2, AGTR1, EDNRB, ATP2B3, PDE1C, PTK2B, PPP3CC, NOS2, PLCB1, CAMK2A, SLC8A1, PTGER3, SPHK2, CCKBR, CACNA1I, HTR4, PTGFR, ITPR3, ITPR1, GNAL, P2RX7, ADRA1B, TBXA2R, ADRA1A, RYR2, GNAS, HTR2C, CACNA1D, ADRA1D, PTAFR, CACNA1A, CACNA1B	6.65×10^{5}
	Vascular smooth muscle contraction: KCNMA1, ADCY2, ADCY8, CALD1, NPR1, ITPR3, ITPR1, AGTR1, ACTG2, PRKCQ, PTGIR, JMJD7-PLA2G4B, PLA2G4A, ADRA1B, ADRA1A, GUCY1A3, GUCY1B3, GNAS, PLCB1, PLA2G2E, CACNA1D, PLA2G4B, ADRA1D, PLA2G2F	1.20×10^{2}

	Cytokine-cytokine receptor interaction: CXCL1, IFNA21, IL1R1, TNF, PDGFB, IL6ST, CXCL3, CRLF2, LEPR, CXCL2, IL13, KITLG, PF4, CXCR2, CXCR3, CXCL11, TNFSF18, TGFB1, IL11, FLT3LG, ACVR1B, IL17A, CCL23, IL17B, CXCR6, IL3, FLT1, IL5, IL7, FLT3, IL9, IL26, CCL16, CCL18, GH2, GH1, CCR7, CCR6, TNFRSF10D, CXCL13, IFNA14	1.17×10^2
	Hematopoietic cell lineage: IL3, IL1R1, IL5, TNF, FLT3, IL7, ITGA1, KITLG, ANPEP, ITGB3, ITGA4, CD1E, IL11, FLT3LG, CD36, GP1BB, CD33, MS4A1, HLA-DRB4	1.19×10^2
	Asthma: FCER1A, IL3, IL5, TNF, PRG2, IL9, HLA-DRB4, IL13, MS4A2, HLA-DOA	1.57×10^2
	Arrhythmogenic right ventricular cardiomyopathy (ARVC): SLC8A1, ITGA1, LEF1, CACNB2, ACTN2, CACNG3, ITGB3, CDH2, ITGA4, TCF7L2, CTNNA3, CTNNA2, ITGB8, DMD, ITGA7, RYR2, CACNA1D	1.67×10^2
	Arachidonic acid metabolism: CYP2U1, TBXAS1, CYP2C18, LTC4S, GPX1, JMJD7-PLA2G4B, PLA2G4A, PTGIS, CYP4F3, GPX7, ALOX5, PLA2G2E, PLA2G4B, HPGDS, PLA2G2F	1.74×10^2
	Dilated cardiomyopathy: SLC8A1, ADCY2, TNF, MYL3, ADCY8, ITGA1, CACNB2, CACNG3, ITGB3, ITGA4, TNNI3, TTN, TGFB1, ITGB8, DMD, ITGA7, RYR2, GNAS, CACNA1D	1.55×10^2
	Fc epsilon RI signaling pathway: PIK3CG, FCER1A, IL3, TNF, IL5, IL13, MAPK11, VAV1, JMJD7-PLA2G4B, PLA2G4A, FYN, MS4A2, INPP5D, PLA2G2E, PLA2G4B, PIK3R1, LCP2, PLA2G2F	1.58×10^2
	Long-term depression: GNAO1, GRIA3, ITPR3, ITPR1, JMJD7-PLA2G4B, PLA2G4A, PPP2CB, GRID2, GUCY1A3, GUCY1B3, GNAS, PLCB1, PLA2G2E, PLA2G4B, CACNA1A, PLA2G2F	3.25×10^2
	Hypertrophic cardiomyopathy (HCM): SLC8A1, TNF, MYL3, ITGA1, CACNB2, CACNG3, ITGB3, ITGA4, TNNI3, TTN, TGFB1, ACE, ITGB8, DMD, ITGA7, RYR2, CACNA1D	3.37×10^2
Cinnamic acid	Retinol metabolism: CYP3A5, CYP3A7, CYP2C19, CYP2C9, CYP2B6, CYP2C8, ADH6, ADH1B, RPE65, ADH7, RDH5, CYP3A43, CYP4A11, RDH8, DHRS3, CYP4A22, LRAT, CYP2A6, UGT2B4	9.71×10^5
	Neuroactive ligand-receptor interaction: F2RL2, CSH1, CCKAR, F2RL3, GRIK1, GABRB2, LEPR, GNRHR, GABBR2, AGTR1, EDNRB, GRIN2B, GALR1, GALR3, GALR2, GRID2, MC5R, CALCRL, HTR1F, CCKBR, GZMA, HTR4, FSHR, CRHR2, CHRM5, ADRB1, GRM8, GPR50, GLP1R, AVPR2, OPRK1, DRD4, FPR1, HCRTR1, APLNR, HRH1, PRSS2, HRH4, ADRA2A, ADRA2B, GABRA1, PTH2R, OPRL1, GABRA5, GRIA3, GH2, GH1, GRIA2, P2RX1	4.87×10^5
	Arachidonic acid metabolism: CYP2U1, TBXAS1, CYP2C19, CYP2C9, CYP2B6, CYP2C8, PTGS1, CYP4A11, JMJD7-PLA2G4B, PLA2G4A, CYP4A22, PTGIS, ALOX15B, GPX7, ALOX5, PLA2G2E, PLA2G4B, PLA2G5, ALOX12, PLA2G2F	5.96×10^5
	Cytokine-cytokine receptor interaction: CXCL1, CSF2, IFNA21, IL1R1, CCL2, TNF, PDGFB, IL6ST, LEPR, CXCL3, BMPR2, CXCR2, PF4, CXCR3, CXCL11, TNFSF18, TGFB1, ACVR1B, IL17A, IFNA1, IL17B, IFNA7, CCL20, CXCR6, CSF2RB, IL4, IL5, IL9, IL26, CD40, CCL18, KDR, CCL11, CCR9, GH2, GH1, CCR7, CCR6, IFNB1, TNFRSF10D, PDGFRB, IL5RA, IFNA13, EDA, MPL, IL3RA	6.42×10^4
	Linoleic acid metabolism: CYP3A43, CYP3A5, PLA2G4A, JMJD7-PLA2G4B, CYP3A7, CYP2C19, CYP2C9, AKR1B10, CYP2C8, PLA2G2E, PLA2G4B, PLA2G5, PLA2G2F	5.47×10^4
	Long-term depression: PRKCA, GNAO1, NOS1, IGF1, GRIA3, ITPR1, JMJD7-PLA2G4B, PLA2G4A, GRIA2, GRID2, GUCY1B3, GNAS, PLCB1, PLA2G2E, PLA2G4B, PLCB2, CACNA1A, PLA2G5, PLA2G2F	2.88×10^3
	Natural killer cell mediated cytotoxicity: CSF2, IFNA21, TNF, KLRC3, KLRK1, CD48, IFNA1, IFNA7, ZAP70, PPP3CC, FCGR3A, FCGR3B, PIK3R1, NFATC1, PRKCA, ICAM1, NCR2, NCR1, FYN, TNFRSF10D, IFNB1, ULBP1, IFNA13, KIR2DL3, KIR3DL1, KIR3DL2, LCP2	2.95×10^3
	Hematopoietic cell lineage: IL4, CSF2, IL1R1, IL5, TNF, FCER2, ITGA1, ANPEP, ITGB3, ITGA4, GP5, CD36, FCGR1C, GP1BB, FCGR1A, MS4A1, HLA-DRB4, CD22, IL5RA, IL3RA, CD7	4.11×10^3
	Drug metabolism: CYP3A5, CYP3A7, CYP2C19, CYP2C9, CYP2B6, CYP2C8, ADH6, ADH1B, ADH7, ALDH3A1, CYP3A43, FMO1, ALDH1A3, AOX1, CYP2A6, UGT2B4	6.05×10^3
	Asthma: FCER1A, CCL11, IL4, IL5, TNF, IL9, HLA-DRB4, MS4A2, HLA-DPB1, CD40	1.28×10^2
	Vascular smooth muscle contraction: PRKCA, KCNMA1, CALD1, NPR1, PRKCE, ITPR1, AGTR1, ACTG2, CYP4A11, JMJD7-PLA2G4B, PLA2G4A, CYP4A22, MYH11, GUCY1B3, GNAS, CALCRL, PLCB1, PLA2G2E, CACNA1D, PLA2G4B, PLCB2, PLA2G5, PLA2G2F	1.48×10^2
	Fc epsilon RI signaling pathway: PRKCA, IL4, FCER1A, CSF2, TNF, IL5, PRKCE, BTK, JMJD7-PLA2G4B, PLA2G4A, FYN, MS4A2, PLA2G2E, PLA2G4B, PIK3R1, PLA2G5, LCP2, PLA2G2F	2.02×10^2
	Metabolism of xenobiotics by cytochrome P450: CYP3A5, CYP3A7, CYP2F1, CYP2C19, CYP2B6, CYP2C9, CYP2C8, ADH6, ADH1B, ADH7, ALDH3A1, CYP3A43, ALDH1A3, UGT2B4	3.05×10^2
	Dilated cardiomyopathy: CACNA2D1, TNF, CACNG5, ITGA1, IGF1, MYH7, MYH6, ITGB3, CACNB4, ITGA4, TTN, TGFB1, ADRB1, ITGB6, SGCD, RYR2, GNAS, CACNA1D	4.02×10^2
	Hypertrophic cardiomyopathy (HCM): CACNA2D1, TNF, CACNG5, ITGA1, IGF1, MYH7, MYH6, ITGB3, CACNB4, ITGA4, TTN, TGFB1, ACE, ITGB6, RYR2, SGCD, CACNA1D	4.04×10^2
	Calcium signaling pathway: CCKAR, ERBB2, ATP2B2, AGTR1, EDNRB, ATP2B3, HRH1, PPP3CC, PLCB1, PLCB2, PRKCA, NOS1, SPHK2, CCKBR, CACNA1I, HTR4, ITPR1, GNAL, CHRM5, ADRB1, CAMK4, P2RX1, RYR3, RYR2, PDGFRB, GNAS, CACNA1D, CACNA1A	4.56×10^2
Fe[illegible]	Neuroactive ligand-receptor interaction: F2RL2, CSH1, F2RL3, GABRB3, GRIK1, GABRB2, TACR1, GLRA3, LEPR, GNRHR, GABBR2, SCTR, EDNRB, PTGIR, GRIN2B, GALR3, GRID2, MC5R, PTGER1, PTGER3, HTR4, FSHR, CRHR2,	4.86×10^6

	SSTR5, GRM8, GRM7, TSHR, GLP1R, C3AR1, AVPR2, CYSLTR1, DRD2, OPRK1, DRD4, BDKRB1, ADRB3, HRH1, PRSS2, MAS1, GABRQ, GABRP, OPRL1, GABRA5, GRIA3, PTGFR, GH1, GPR35, P2RX7, P2RY11, GRIA2, TBXA2R, ADRA1A	
	Hematopoietic cell lineage: IL3, IL9R, CR2, IL5, FLT3, CSF1, GYPA, ITGA1, MME, ANPEP, IL6R, ITGB3, ITGA4, IL11RA, FLT3LG, GP1BB, IL1B, TPO, IL5RA, CD5, CSF2RA, IL3RA, CD7	4.06×10^4
	Cytokine-cytokine receptor interaction: IL9R, PDGFB, CSF1, LEPR, CXCL3, CXCL2, GDF5, CXCR2, PF4, CXCR3, TNFRSF4, TNFSF18, TGFB1, FLT3LG, ACVR1B, TNFRSF1B, IL17B, IFNA7, IFNA4, IL1B, TPO, IL2RG, FAS, RTEL1, CSF2RA, IL3, IL5, FLT1, FLT3, IL26, CD40, IL6R, CCL16, IL11RA, CCL17, CCL11, CCR9, GH1, TNFSF10, CCR6, CCR5, TNFRSF10D, IL5RA, EDA, IL3RA	1.75×10^3
	Arachidonic acid metabolism: CYP2U1, CYP2C19, CYP2C8, GPX1, CYP4A11, JMJD7-PLA2G4B, CYP4A22, PTGIS, PTGDS, ALOX15B, PLA2G1B, ALOX5, PLA2G2E, PLA2G4B, PLA2G5, ALOX12, PLA2G2F	3.76×10^3
	ECM-receptor interaction: TNC, ITGA1, ITGB3, ITGA4, COL5A3, COL5A1, COL4A6, VWF, LAMB4, LAMA4, GP1BB, ITGB8, ITGA7, COL1A2, COL6A2, COL6A1, SV2A, LAMB1, THBS1	1.35×10^2
	Focal adhesion: PDGFB, TNC, ELK1, ITGB3, SRC, CDC42, LAMB4, PAK7, ITGB8, COL6A2, COL6A1, ZYX, LAMB1, THBS1, SHC3, PIK3CG, FLT1, ITGA1, ACTN2, ACTN3, ITGA4, FLNC, COL5A3, VAV1, COL5A1, COL4A6, PRKCB, VWF, LAMA4, CCND2, FYN, ITGA7, COL1A2, PARVB	1.34×10^2
	Natural killer cell mediated cytotoxicity: PIK3CG, ICAM1, CD244, HLA-C, NCR2, NCR1, VAV1, PRKCB, CD48, SH2D1A, TNFSF10, IFNA7, FYN, TNFRSF10D, ULBP1, LCK, IFNA4, ZAP70, NFATC4, FAS, KIR2DL3, SHC3, KIR3DL1, LCP2, KIR3DL2	1.72×10^2
	Long-term depression: GNAO1, GRIA3, ITPR3, PRKCB, JMJD7-PLA2G4B, GRIA2, CRH, GRID2, PLA2G1B, GUCY1B3, GNAS, PLCB1, PLA2G2E, PLA2G4B, PLCB2, PLA2G5, PLA2G2F	2.16×10^2
	GnRH signaling pathway: ADCY2, GNRH2, ELK1, MAPK11, GNRHR, MMP14, ITPR3, SRC, PRKCB, CDC42, JMJD7-PLA2G4B, PLA2G1B, GNAS, PLCB1, PLA2G2E, PLA2G4B, CAMK2A, PLCB2, PLA2G5, MAP2K6, PLA2G2F	1.94×10^2
	Fc epsilon RI signaling pathway: PIK3CG, FCER1A, IL3, IL5, MAPK11, PRKCE, VAV1, BTK, PRKCB, JMJD7-PLA2G4B, FYN, PLA2G1B, PLA2G2E, PLA2G4B, MAP2K6, PLA2G5, LCP2, PLA2G2F	2.30×10^2
	Cell adhesion molecules (CAMs): PVR, CLDN8, ICAM1, CADM3, HLA-C, NRXN1, ITGA4, CDH2, CD40, CLDN11, CDH4, PDCD1, SIGLEC1, CDH15, PVRL1, ITGB8, ICOS, PECAM1, CNTNAP1, VCAN, JAM2, HLA-DOA, ICOSLG, CD28	2.30×10^2
	Linoleic acid metabolism: CYP3A4, JMJD7-PLA2G4B, CYP3A7, CYP2C19, CYP2C8, PLA2G1B, PLA2G2E, PLA2G4B, PLA2G5, PLA2G2F	3.57×10^2
Caffeic acid	Neuroactive ligand-receptor interaction: F2RL2, CSH1, F2RL3, THRA, GRIK1, GABRB2, GLRA3, GRIK3, LEPR, GRIK4, GNRHR, AGTR1, PTGIR, GRIN2B, NMUR1, GALR3, GALR2, HTR1D, PTGER1, GZMA, FSHR, CRHR1, CRHR2, GRM4, ADRB1, GRM7, PTGDR, GPR50, GLP1R, AVPR2, DRD1, DRD3, DRD2, NPY2R, FPR3, HCRTR1, HRH1, PRSS2, ADRA2A, ADRA2B, GABRA2, OPRL1, GRIN1, PTGFR, GH2, GH1, GPR35, AVPR1B, ADRA1B, ADRA1A, HTR2C, HTR2A	1.34×10^6
